# Salvage Radical Hysterectomy for Recurrent Cervical Cancer in a Previously Irradiated Field Using the hinotori™ Surgical Robot System: A Case Report

**DOI:** 10.7759/cureus.96395

**Published:** 2025-11-08

**Authors:** Seiji Mabuchi, Naoko Komura, Yu Wakimoto, Tomoko Ueda, Hiroshi Tsubamoto

**Affiliations:** 1 Department of Obstetrics and Gynecology, Hyogo Medical University, Nishinomiya, JPN; 2 Department of Obstetrics and Gynecology, Kaizuka City Hospital, Kaizuka, JPN

**Keywords:** cervical cancer, hinotori surgical robot system, irradiated field, recurrence, robot-assisted surgery, salvage hysterectomy

## Abstract

Salvage radical hysterectomy for recurrent cervical cancer within a previously irradiated field is technically demanding due to radiation-induced fibrosis and adhesions. Robot-assisted surgery may offer advantages over open surgery, including enhanced three-dimensional visualization, improved ergonomics, and tremor filtration, potentially enabling safer and more precise dissection in fibrotic tissue planes. However, to date, evidence in the recurrent setting remains limited and has been reported exclusively with the da Vinci® Surgical System (Intuitive Surgical, Sunnyvale, California, United States). In this report, we describe a case of salvage radical hysterectomy performed with the hinotori™ Surgical Robot System (Medicaroid Corporation, Kobe, Japan) in a 60-year-old woman with locally recurrent cervical cancer in a previously irradiated field. Despite the technical difficulties associated with operating in a previously irradiated field with radiation-related fibrosis, the recurrent cervical tumor was completely resected without intraoperative or postoperative complications. This case demonstrates the potential feasibility and safety of hinotori-assisted salvage radical hysterectomy for complex procedures in irradiated fields and warrants further evaluation in larger series.

## Introduction

Concurrent chemoradiotherapy (CCRT) remains the standard primary treatment for locally advanced cervical cancer. However, approximately one-third of patients experience disease recurrence within three years after completing primary therapy [1]. Previous investigations have suggested that recurrence is localized to the pelvis in approximately 40% of patients who have undergone prior definitive radiotherapy [2]. 

Managing recurrent disease within a previously irradiated field presents a significant clinical challenge. Curative options in this setting are largely limited to re-irradiation and surgery because chemotherapy has been ineffective in this patient population [3]. While re-irradiation has been used sparingly due to the high incidence of severe late toxicity [4], salvage surgery has demonstrated survival benefits, with reported five-year survival rates ranging from 30% to 60% [5-10]. As a surgical salvage procedure, pelvic exenteration (PE) has historically been the most frequently performed approach. However, PE is highly invasive and invariably necessitates stoma creation. As a less invasive alternative, salvage hysterectomy, including extrafascial hysterectomy or radical hysterectomy, may be considered for patients with centrally recurrent or persistent cervical cancer confined to the pelvis [11]. Nevertheless, in regions previously exposed to high-dose radiation, where marked fibrosis, dense adhesions, and tissue sclerosis are present, it remains technically demanding to achieve complete uterine resection with curative intent while preserving bladder and rectal function without injury. To date, most salvage hysterectomies have been performed via an open surgical approach [7]. Reported favorable prognostic factors include negative surgical margins, limited tumor size, and the absence of nodal involvement [12-14].

In recent years, robot-assisted surgery has emerged as a minimally invasive alternative to open surgery in cancer treatment, including gynecologic cancer. However, at least in the field of gynecologic malignancies, data on its feasibility and oncological outcomes in the recurrent setting remain limited. Several reports have suggested that robot-assisted salvage radical hysterectomy (RH) [15] or pelvic exenteration [16,17] may be feasible, safe, and oncologically effective for recurrent cervical cancer arising in previously irradiated fields. However, all such procedures have been performed using the da Vinci® Surgical System (Intuitive Surgical, Sunnyvale, California, United States; hereafter referred to as da Vinci).

The hinotori™ Surgical Robot System (Medicaroid Corporation, Kobe, Japan; hereafter referred to as hinotori) was developed as a domestically produced, cost-effective alternative to existing robotic platforms. It received regulatory approval in Japan for urologic surgeries in 2020, and subsequently for gynecologic and gastrointestinal indications in late 2022 [18]. In the field of gynecology, the hinotori is currently approved by Japan’s National Health Insurance for use in the treatment of benign uterine tumors and stage I endometrial cancer [19]. To our knowledge, there have been no previous reports documenting the feasibility or surgical outcomes of hinotori-assisted salvage RH.

Here, we present a case of salvage RH using the hinotori for recurrent cervical cancer occurring within a previously irradiated field.

## Case presentation

A 60-year-old postmenopausal Japanese woman, gravida 4 para 3, was referred to our hospital for surgical management of locally recurrent cervical cancer. She had no significant medical or surgical history aside from a prior diagnosis of International Federation of Gynecology and Obstetrics (FIGO) stage IIB cervical adenocarcinoma.

Twenty-eight months before her referral to our hospital, a bulky cervical tumor with invasion of the posterior vaginal fornix and parametrial tissue was identified at a regional hospital. Following a full pretreatment evaluation, she was diagnosed with FIGO stage IIB cervical cancer and underwent concurrent chemoradiotherapy (CCRT), which included external-beam radiotherapy (45 Gy in 25 fractions) with weekly cisplatin (40 mg/m²), followed by high-dose-rate brachytherapy (25 Gy in five fractions).

Two months after completing CCRT, her serum CA125 level had increased to 44.4 U/mL, compared to 17.3 U/mL at the conclusion of treatment. Although cervical cytology did not reveal residual disease, 18F-fluorodeoxyglucose positron emission tomography/computed tomography (FDG-PET/CT) showed intense FDG uptake confined to the uterine cervix, with no evidence of distant disease. Based on these findings, a diagnosis of locally recurrent cervical cancer was made.

**Figure 1:**
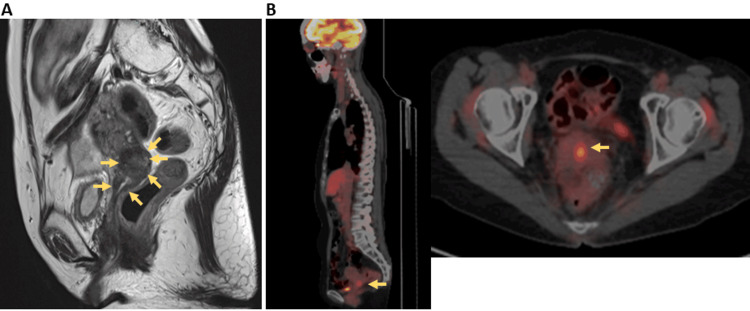
Imaging of recurrent cervical cancer. (A) Sagittal T2-weighted MRI. The yellow arrows indicates a cervical mass extending to the upper third of the vagina, suggesting recurrent cervical cancer. (B) FDG-PET/CT. The yellow arrow highlights increased FDG uptake in the cervix, consistent with solitary recurrent cervical cancer. FDG-PET/CT: 18F-fluorodeoxyglucose positron emission tomography/computed tomography

She subsequently received six cycles of combination chemotherapy with paclitaxel, cisplatin, and bevacizumab. A complete response was achieved, and she continued with maintenance bevacizumab therapy. However, 20 months after the initial recurrence, while still on maintenance therapy, her CA125 level began to rise again. A cervical smear revealed adenocarcinoma. Pelvic magnetic resonance imaging (MRI) demonstrated a 4.0-cm contrast-enhancing mass in the uterine cervix (Figure 1A). FDG-PET/CT once again showed significant FDG uptake limited to the cervix (Figure 1B), confirming a second local recurrence. At the patient’s request for surgical intervention, she was referred to our institution. After multidisciplinary discussion and detailed consultation with the patient and her family, robot-assisted RH with bilateral salpingo-oophorectomy was planned and performed.

Surgical procedure

To minimize the risk of ureteral injury, bilateral double-J ureteral stents were placed preoperatively. Robot-assisted surgery was performed using the hinotori. Due to fibrosis and rigidity of the vaginal tissue resulting from prior radiation therapy, creation of a vaginal cuff was deemed unfeasible and thus omitted.

**Figure 2:**
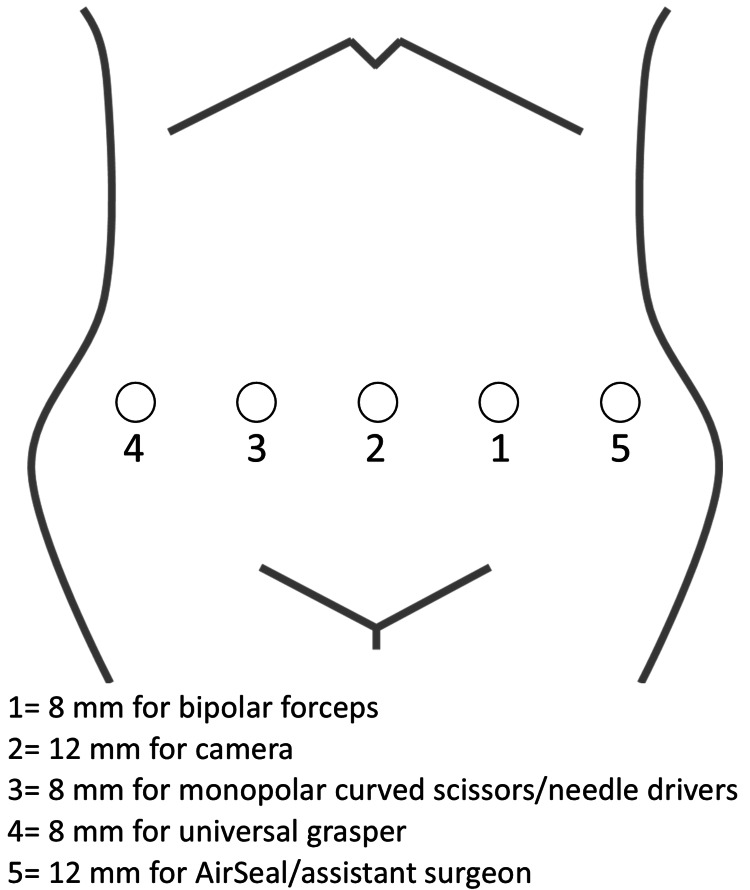
Positioning of the trocars. Four robotic trocars were placed 7 cm apart at the umbilical level, and an assist port was positioned at the far-left lateral site. Image Credit: Authors

Four robotic trocars were placed 7 cm apart at the umbilical level, and an assist port was positioned at the far-left lateral site (Figure 2). A 12-mm trocar was used for the endoscope, while three 8-mm robotic ports and a 5-mm assistant port were placed sequentially. After port placement, the patient was positioned in a 20° Trendelenburg tilt, and the hinotori operating unit was docked. The surgical instruments used included bipolar fenestrated forceps through the first 8-mm port, monopolar curved scissors and a wide needle holder through the third 8-mm port, and a universal grasper through the fourth 8-mm port. A zero-degree endoscope was utilized throughout the procedure.

To prevent the spillage of tumor cells into the peritoneal cavity, the bilateral fallopian tubes were ligated using surgical clips. The round ligaments were transected, and the anterior leaf of the broad ligament was opened. The paravesical and pararectal spaces were developed via blunt dissection. The bilateral uterine arteries and ureters were then identified. The uterine arteries were ligated and transected at their origins. After identifying the cardinal ligaments, the ureters were carefully dissected free from the retroperitoneal tissues and uterine vasculature, and retracted with silicone slings, as previously described [20]. The anterior and posterior vesicouterine ligaments were then transected. The infundibulopelvic ligaments were isolated, coagulated, and transected. The uterosacral ligaments were divided, and the rectum was carefully separated from the posterior vaginal wall. The remaining connective tissues surrounding the vagina were excised, and the uterus was detached. The surgical specimen was extracted transvaginally.

The procedural steps for salvage RH were largely similar to those previously reported using the da Vinci [15]. Throughout the procedure, a vaginal uterine manipulator was not used. Instead, uterine manipulation was achieved using the U-traction device, as previously described [21]. To enhance surgical exposure, a Vagi-Pipe® (Hakko Co., Ltd., Nagano, Japan) was inserted transvaginally.

Intraoperative inspection revealed that the uterus had descended into the retroperitoneal space due to prior radiation, while the bladder appeared relatively elevated, resulting in markedly reduced uterine mobility. Despite the technical difficulties associated with operating on previously irradiated, fibrotic tissue, the recurrent cervical tumor was completely resected using the hinotori. The procedure was completed without intraoperative complications. Total operative time was 287 minutes, with no measurable blood loss and no need for blood transfusion.

Following hysterectomy, thorough pelvic lavage was performed with the dual purpose of recovering any cancer cells that might have been shed intraoperatively and of reducing bacterial contamination from the vaginal tract to minimize the risk of postoperative infection. In accordance with the institutional protocol, a urethral catheter was maintained for six days postoperatively to preserve bladder function. No postoperative complications occurred.

**Figure 3:**
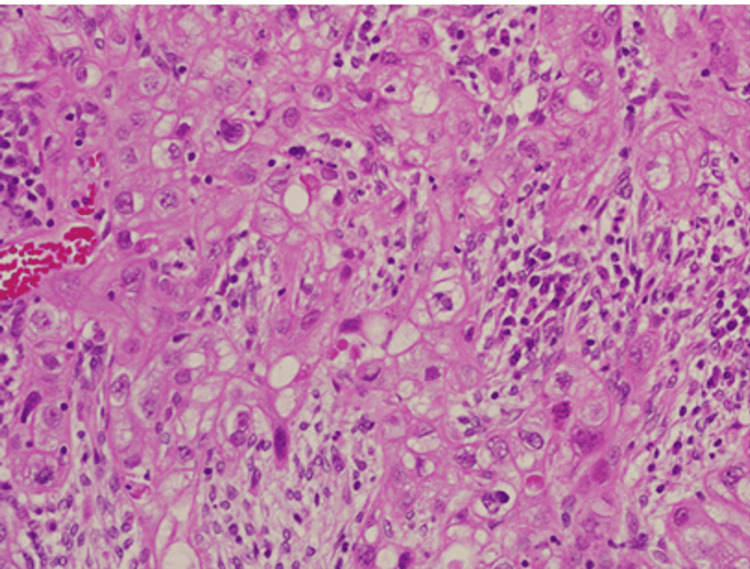
Hematoxylin and eosin staining of the lesion showing poorly differentiated carcinoma (x 400)

Pathological examination of the surgical specimen confirmed a 4.0-cm poorly differentiated carcinoma consistent with recurrent cervical cancer (Figure 3), which was resected en bloc with the surrounding tissues, and the surgical margin was 6 mm. No adjuvant therapy was administered after surgery. Postoperatively, the patient was followed up every two to three months in the outpatient clinic. At each visit, tumor marker tests, a Papanicolaou (Pap) smear, and transvaginal ultrasonography were performed. A chest and abdominal CT scan was obtained six months after surgery, which showed no abnormal findings. At 10 months postoperatively, the patient remains alive with no evidence of disease.

## Discussion

In this report, we present a successful case of hinotori-assisted salvage RH for recurrent cervical cancer in a previously irradiated field. Our experience demonstrates the utility of the hinotori in such a technically challenging procedure.

A recent systematic review involving 601 patients with locally recurrent cervical cancer who underwent open salvage RH reported a positive surgical margin rate of 17.6% and a severe postoperative complication rate (grade ≥3) in 29.8% of cases [22]. Other studies on pelvic exenteration or RH have also reported high morbidity rates (30-60%) [5-10]. These reports strongly suggested that salvage RH following radiotherapy is technically highly challenging due to radiation-induced fibrosis and adhesions in the vesicouterine, vesicovaginal, and rectovaginal spaces. In this context, robot-assisted surgery may offer advantages over open surgery, including enhanced three-dimensional visualization, improved ergonomics, and tremor filtration, potentially enabling safer and more precise dissection in fibrotic tissue planes. In our recent publication comparing open salvage RH and robot-assisted RH using the da Vinci Xi, we have shown that robot-assisted surgery significantly reduced blood loss, while the rates of intraoperative and postoperative complications were comparable between groups [15]. Kaplan-Meier analyses for progression-free and overall survival also showed no significant differences. The present case, in which the hinotori was employed, along with a few previous reports employing the da Vinci [15-17], may indicate the usefulness of a robot-assisted approach in this patient population.

The hinotori has several unique features compared to the da Vinci. It is equipped with independently controlled eight-axis arms, which allow for more natural, human-like movements and improved instrument maneuverability in tight pelvic spaces. In addition, its docking-free design maintains a wider operative field around the trocars, which facilitates better instrument handling and smooth transitions to conventional laparoscopy if necessary. Furthermore, the system is more compact and cost-effective, potentially improving accessibility for small and mid-sized institutions [23]. Therefore, if this surgical assistance robot becomes widely used for many diseases and surgical procedures, it should benefit many patients. Salvage RH is one example, but it is unclear whether the hinotori can be applied to difficult surgeries such as pelvic exenteration and para-aortic lymph node dissection. We hope that the utility of the hinotori in such advanced procedures will be verified in the near future.

In addition to the technical aspects, our case offers clinical insights into post-treatment surveillance. While CA-125 is not routinely recommended for surveillance in cervical cancer, it may be informative in cervical adenocarcinoma. Recent studies suggest that CA-125 levels ≥50 U/mL are associated with poorer prognosis and may be useful for detecting disease recurrence in patients with surgically resected cervical adenocarcinoma [24]. In our patient, a rising CA-125 during follow-up prompted imaging that confirmed local recurrence, indicating its potential utility as a useful marker for disease monitoring. Similarly, cervicovaginal cytology after radiotherapy has limited sensitivity but a high positive predictive value and may precede clinical symptoms in detecting local recurrence after radiotherapy [25,26]. Detecting recurrent disease through cervical biopsy is also challenging because the cervical surface that received the highest radiation dose during intracavitary brachytherapy-where biopsy is technically easiest, usually shows no residual tumor, whereas recurrent lesions tend to develop deeper within the cervical tissue. Therefore, it is essential to employ multiple diagnostic modalities, including imaging studies and tumor marker assessments, to achieve an accurate diagnosis and facilitate the early detection of recurrent cervical cancer. In our case, cytology suggesting adenocarcinoma, together with a rising CA-125, supported the imaging findings and contributed to confirming local recurrence.

Although our case suggests the safety and utility of the hinotori in patients with locally recurrent cervical cancer, we do not recommend the use of a minimally invasive surgery (MIS) approach for recurrent cervical cancer patients without reflecting on the results of the laparoscopic approach to cervical cancer (LACC) trial that demonstrated the negative impact of minimally invasive surgery on oncologic outcomes in cervical cancer management [27]. As the LACC trial was conducted in patients with newly diagnosed cervical cancer, not with locally recurrent cervical cancer, the role of MIS in all patients with recurrent cervical cancer remains undetermined. Although our previous experiences have suggested the utility of a robot-assisted approach for locally recurrent cervical cancer without compromising patient survival [15], there are very few reports on robot-assisted surgery in this area. In addition, although there are some reports suggesting the usefulness of laparoscopic surgery, these are case series with a small number of cases [28]. Because sufficient clinical evidence has not been established, we believe that the robotic approach should be employed cautiously with appropriate patient counseling.

## Conclusions

We reported a case of salvage RH for recurrent cervical cancer in a previously irradiated field utilizing the hinotori. The operation was completed with negative margins and without intraoperative or postoperative complications. This case suggests that a hinotori-assisted approach may be a feasible option for complex salvage procedures in irradiated fields. Further studies in larger series are warranted to clarify the safety, effectiveness, and oncologic outcomes in patients with locally recurrent cervical cancer.
